# Distal radius fractures in children: substantial difference in stability between buckle and greenstick fractures

**DOI:** 10.3109/17453670903316850

**Published:** 2009-10-01

**Authors:** Per-Henrik Randsborg, Einar A Sivertsen

**Affiliations:** Department of Orthopedic Surgery, Akershus University HospitalNorway

## Abstract

**Background and purpose** Numerous follow-up visits for wrist fractures in children are performed without therapeutic consequences. We investigated the degree to which the follow-up visits reveal complications and lead to change in management. The stability of greenstick and buckle fractures of the distal radius was assessed by comparing the lateral angulation radiographically.

**Patients and methods** The medical records of 305 distal radius fractures in patients aged less than 16 years treated at our institution in 2006 were reviewed, and any complications were noted. The fracture type was determined from the initial radiographs and the angulation on the lateral films was noted.

**Results** Only 1 of 311 follow-ups led to an active intervention. The greenstick fractures had more complications than the buckle fractures. The lateral angulation of the buckle fractures did not change importantly throughout the treatment. The greenstick fractures displaced 5° on average, and continued to displace after the first 2 weeks. On average, the complete fractures displaced 9°.

**Conclusion** Buckle fractures are stable and do not require follow-up. Greenstick fractures are unstable and continue to displace after 2 weeks. Complete fractures of the distal radius are uncommon in children, and highly unstable. A precise classification of fracture type at the time of diagnosis would identify a smaller subset of patients that require follow-up.

## Introduction

Distal radius fractures are the most common fracture in childhood (Landin 1997), and the incidence is rising (Hagino et al. 2000, Khosla et al. 2003). Most minimally displaced fractures are treated without manipulation, and immobilized between 3 and 6 weeks. Displaced fractures are often manipulated before immobilization. The rate of long-term complications in childhood distal radius fractures is low. Despite this, clinical and radiographic follow-up examinations are frequently performed (Green et al. 1998). The great remodeling potential in a child's distal radius allows dorsal angulation of up to 20° for good clinical and anatomical long-term results (Friberg 1979, Qairul et al. 2001).

In childhood, the periosteal sleeve is thick and protects the cortex. The bone is softer and more pliable than in adults. This accounts for the range of different fracture types that is uniquely seen in childhood: the buckle (torus), the classical greenstick fracture, the complete fractures (adult type), and the fractures involving the growth plate. In addition, the plasticity of the children's long bones can cause a bowing of the radius.

Many authors consider buckle fractures to be stable (Farbman et al. 1999, Symons et al. 2001, Solan et al. 2002), but one study reported 7% subsequent displacement among buckle fractures (Schranz and Fagg 1992). Greenstick fractures are less stable. The periosteal hinge has been considered important for the stability of fractures (Zamzam and Khoshhal 2005). Complete fractures are therefore considered highly unstable. Physeal fractures can result in growth disturbances (Cannata et al. 2003), and are often monitored closely. Follow-up radiographs are undertaken to identify fractures that become displaced and need manipulation with or without Kirschner-wire fixation.

We investigated the degree to which the clinical and radiographic follow-ups reveal complications and lead to a change in management of the unmanipulated distal radius fractures in children less than 16 years of age. We determined the frequency and type of complications registered during treatment, and assessed the stability of the different fracture types.

## Patients and methods (Figure 1)

In 2006, 77,470 individuals under the age of 16 years lived in the catchment area of Akershus University Hospital, which is north-east of Oslo, Norway. We used our institution's database to identify all patients below the age of 16 years who presented at our institution in 2006 with a fracture of the distal radius. Fractures not involving the metaphysis according to the AO Pediatric Comprehensive Classification of long bone fractures were considered to be diaphyseal fractures, and were excluded from the study (Slongo et al. 2006).

**Figure 1.**
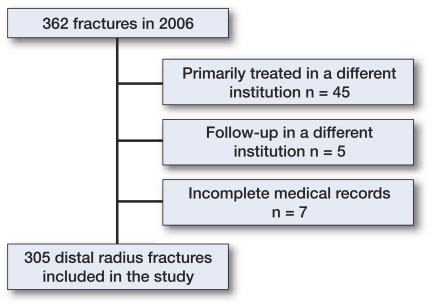
Flow diagram of patients less than 16 years old with a distal radius fracture who were treated at our institution in 2006. Exclusion criteria are shown to the right.

362 patients were identified. This represents an incidence of 47 distal radial fractures per 10,000 children per annum. We excluded 50 children who had been treated in part at other hospitals, as treatment protocols might vary between hospitals, and follow-up strategy could be different for transit patients. 7 cases with missing radiographs were also excluded. We then retrospectively reviewed the medical records and extracted the relevant data: date of injury and date of initial presentation, the type and length of immobilization, any complications, and clinical and radiographic follow-up visits.

265 fractures were treated without manipulation and 40 fractures were treated with closed or open reduction.

### Radiographic analysis

**Figure 2.**
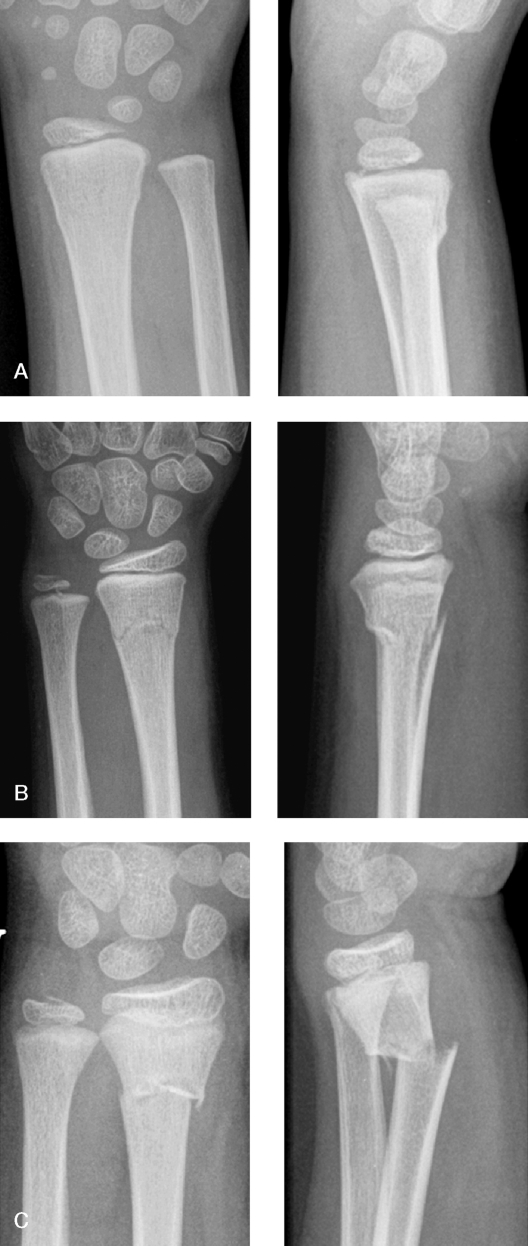
Primary radiographs of 3 different metaphyseal fractures, demonstrating the difference in cortical involvement in buckle (A), greenstick (B), and complete (C) fractures. A. Buckle fracture in a 7-year-old girl. B. Greenstick fracture in an 8-year-old girl. C. Complete fracture in a 7-year-old boy.

Standard digitized anteroposterior and lateral radiographs were used in this study. All radiographs were reviewed by the investigators. The fractures were classified as buckle (torus) if there was a compression failure of bone without disruption of the cortex on the tension side of the bone, greenstick if the cortex was disrupted on the tension side, and complete if both cortices were disrupted in one projection (Figure 2). Fractures involving the physis were assigned according to the Salter-Harris classification. Buckle fractures were most common (Table 1).

**Table 1.** Distribution of fracture types

Fracture type		Primarily manipulated n = 40	Unmanipulated n = 265
Physeal fractures (n = 32)		21	11
Salter Harris	I	2	2
	II	19	8
	III	–	1
Metaphyseal fractures (n = 273)		19	254
Buckle		1	207
Greenstick (unicortical)		10	34
Complete (bicortical)		8	9
Other (unclassifiable)		–	4

The angle of the physis on the radial axis (the epiphyseal axis angle) was measured on the lateral films as previously described (Lautman et al. 2002). Normally, this angle is 90 degrees. To assess stability of the fractures, the angulation on presentation was compared with the angulation on all later radiographs until plaster removal.

### Statistics

We used SPSS software. Unless otherwise indicated, t-test was used to compare continuous variables in groups. Categorical data were analyzed using chi-square test. Probabilities of less than 0.05 were considered significant.

## Results

### The sex ratio differed between groups

172 (56%) of the 305 children were boys. 147 of 265 unmanipulated fractures and 25 of 40 manipulated fractures occurred in boys. There were 20 boys and 12 girls with physeal fractures.

### Difference between unmanipulated and manipulated fractures

The unmanipulated fractures exhibited less angulation on the lateral film than the manipulated fractures. On presentation, the mean lateral angulation for the unmanipulated fractures was 4.7° (SD 4.7) and 19° (SD 11) for the manipulated fractures (p < 0.001). Although the patients with unmanipulated metaphyseal fractures were on average a year younger than the patients with manipulated metaphyseal fractures (10.9 (5.4–14) vs. 9.8 (1.3–16) years), this difference was not statistically significant (p = 0.2). There were more complete fractures in the unmanipulated group (p < 0.001). Only 1 buckle fracture (of 208) was manipulated.

### Patients with physeal fractures were older and more prone to being manipulated

The patients with a fracture involving the physis (n = 32, 10%) were older than the rest of the study population (12.1 (6.3–16) vs. 9.9 (1.3–16) years, p < 0.001) and had a relative risk of 9 of being manipulated compared with other fracture types.

### Buckle fractures constitute the majority of the unmanipulated fractures and are extensively controlled, clinically as well as radiologically

Manipulated fractures and epiphyseal fractures need to be monitored closely. We therefore excluded all the manipulated fractures, as well as the unmanipulated physeal fractures, from the following analyses. There were 254 unmanipulated metaphyseal fractures, 138 in boys (54%). The left side was involved in 147 (58%) of cases. Mean age was 9.8 (1.3–16) (SD 3.4) years. A follow-up appointment was made in 84% of cases. The mean immobilization time in plaster was 24 (10–41) (SD 5.5) days. There were over 350 follow-up radiographic examinations and 311 clinical follow-ups. The most common fracture was the buckle (n = 207), representing 81% of the unmanipulated fractures, while only 9 fractures (4%) were complete.

### Childhood distal radius fractures show a low frequency of complications

Only 17 complications were registered in our medical records, demonstrating the benign nature of these fractures (Table 2). Only 1 patient had a severe complication. This was a complete fracture with an initial dorsal angulation of 12°, which displaced to 36° after 2 weeks, and was subsequently reduced and pinned. There were more complications among the greenstick fractures than among the buckle fractures (p < 0.001). Furthermore, there were only mild complications in the buckle group.

**Table 2.** Distribution of complications: unmanipulated metaphyseal fractures. The mild complications included transient complaints such as pressure sores, tenderness, and stiffness from the plaster. Moderate complications included clinical deformity and/or radiological malunion at the end of treatment. Severe complications were defined as surgical intervention

Type of complication	Torus/Buckle	Greenstick	Complete	Total
Severe	–	–	1	1
Moderate	–	6	3	9
Mild	6	1		7
Ratio within group	6/207	7/34	4/9	

### Buckle fractures are stable, whereas greenstick fractures show a more unpredictable course

**Figure 3.**
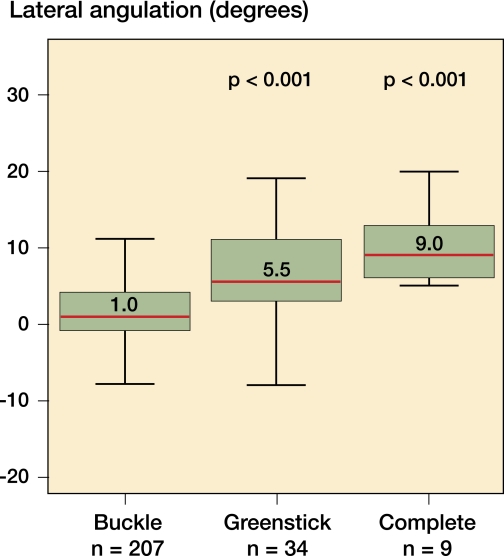
Box plot showing distribution of lateral angulation at presentation by fracture type among unmanipulated radius fractures. Median value is shown in each box. The p-values relate to median lateral angulation against the median for buckle fractures (Mann-Whitney test).

To investigate the stability of the fractures, the epiphyseal axis angle was measured on the lateral films at presentation (Figure 3) and at subsequent follow-ups. We identified the fractures that had a follow-up radiograph taken within 2 weeks and on the day of plaster removal, and compared the epiphyseal axis angle with the radiographs taken at presentation (Table 3).

**Table 3.** Angular stability within 2 weeks and at the time of plaster removal. Change in epiphyseal axis angle measured on the lateral film on presentation and at first radiological follow-up within 14 days (mean 8.6 days) (A) and at the time of plaster removal (B). Worsening of the original displacement is given a positive value for both volar and dorsal fractures, while change towards the anatomical epiphyseal axis angle is negative

Fracture type	No. of patients	Angular change		95% CI	p-value
		mean	SD		
A. Buckle/Torus	44	0.4°	2.9	-0.4–1.3	0.3
Greenstick	20	1.8°	3.5	0.1–3.5	0.03
Complete	9	2.7°	6.1	-2.0–7.4	0.2
B. Buckle/Torus	42	0.9°	3.1	-0.04–1.9	0.06
Greenstick	19	5.1°	6.9	1.8–8.4	0.005
Complete	8	9.0°	10.0	0.7–17.3	0.04

**Figure 4.**
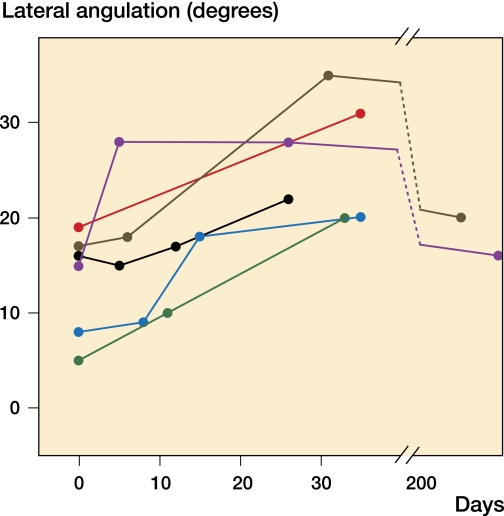
Graph showing the change in lateral angulation over time for the 6 greenstick fractures that ended up with a dorsal angulation of over 20°. 2 fractures had a follow-up after 6 months, demonstrating remodeling potential. One fracture improved at first follow-up, but then displaced to end up over 20°.

Of the 6 greenstick fractures that ended up with a dorsal angulation of more than 20º on the lateral film, only 1 had a follow-up radiograph that indicated how the fracture would end up (Figure 4).

## Discussion

Most of the unmanipulated fractures in our material were minimally displaced and healed without complication or need for secondary intervention. The majority of fractures were stable and did not need follow-up. No complications in the buckle or greenstick group led to surgical intervention. Of more than 300 clinical follow-up examinations, only 1 led to change in management. A precise classification of distal radius fractures in children will facilitate a more efficient follow-up strategy to identify unstable fractures. We have not found any reason to believe that the practice in the current cohort differs substantially from other hospitals in the western world, as similar results have been published by others (Green et al. 1998, Stoffelen and Broos 1998, Jones et al. 2000, Al-Ansari et al. 2007, Hove and Brudvik 2008).

Like others, we found that buckle fractures are stable (Farbman et al. 1999, Solan et al. 2002). The buckle fractures with a follow-up radiograph (n = 75) were more displaced, in terms of angulation in the lateral view, than the rest of the buckle fractures (p < 0.001). This indicates that even the most displaced buckle fractures are inherently stable fractures. Treatment is aimed at comfort, reassurance, and patient and parent satisfaction. Some authors believe one should just splint these fractures and leave it to the general practitioners to follow them up (Davidson et al. 2001). It has also been argued that since these fractures are so benign, casting can cause more harm than good (Plint et al. 2004). We noted some transient complains from pressure sores and stiffness due to casting. We see no need to cast these fractures. It has been shown that removable bracing promotes earlier functional recovery than a full cast (West et al. 2005, Plint et al. 2006). We now use a dorsal slab made of conventional plaster, which can easily be removed by the parents after 3 weeks. A prefabricated removable splint could also be used (Davidson et al. 2001, Solan et al. 2002, Plint et al. 2006).

Greenstick fractures are, however, unstable. Moreover, they continue to displace also after the first 2 weeks. None of the unmanipulated greenstick fractures in our material were subsequently manipulated, despite multiple radiographic controls. If the controls never lead to a change in management, it can be argued that follow-ups for unmanipulated greenstick fractures are unnecessary. The remodeling potential is great in the distal radius of children, and even dorsal angulation over 20º can remodel completely (Fuller and McCullough 1982, Johari and Sinha 1999, Wilkins 2005). It is therefore argued that dorsal angulation can be accepted (Al-Ansari et al. 2007). Excellent long-term functional and anatomical results have been reported (Hove and Brudvik 2008). However, even though a deformed wrist in childhood will remodel over time, it is unknown what consequences the transient deformity will have with regard to physical development and participation in activities. In our department, a dorsal angulation of less than 20 degrees is accepted if the patient has more than 2 years of growth left. Our study shows that the greenstick fractures are unstable, and it is difficult to identify which fractures will displace beyond 20º. We advocate addressing a proper 3-point pressure plaster to help maintain the position in the immobilization period. A bent plaster makes a straight bone (Alemdaroglu et al. 2008).

Brudvik and Hove (2003) reported an annual incidence of 66 distal radius fractures per 10,000 children who were less than 16 years old in Bergen, Norway. It might be that the retrospective nature of our study led to underestimation of the fracture incidence, as we could have lost some fractures to neighboring hospitals. Fractures given a wrong ICD-10 code (different from S52.5, International Statistical Classification of Diseases and Related Health Problems, tenth revision) will also have been lost to our study. However, the coding of fractures is closely related to the financial reimbursement of the hospital, and checked monthly. The low annual incidence of 47 per 10,000 children less than 16 years old in our study is consistent with the results of Kopjar and Wickizer (1998), who reported an overall annual fracture incidence of 128 per 10,000 children less than 12 years old in Rogaland, Norway .

Brudvik and Hove (2003) reported that 11% of distal radius fractures in children were manipulated. This is consistent with our results (13%). They also found that more boys than girls sustained a distal radius fracture (59%), which is similar to our results (56%). The same authors found 19% physeal fractures in their population, as compared to 10% in our material.

Distal radius fractures in childhood have few complications, and most are transient. We have not re-examined our patients, however, and our information is based on retrospective review of the medical records from a single institution. Some patients with complications might have contacted other hospitals or remained at home. Although a moderate degree of underestimation of mild and moderate complications is possible, nearly all severe complications with need of intervention are treated at our institution. Thus, our results support the notion that wrist fractures in childhood are benign. There is much to be gained if treating physicians distinguish between buckle and greenstick fractures. Most wrist fractures in children are stable buckle fractures that do not require follow-up.
